# Antinociceptive Activity and Toxicity Evaluation of the Fatty Oil from *Plukenetia polyadenia *Mull. Arg. (Euphorbiaceae)

**DOI:** 10.3390/molecules20057925

**Published:** 2015-04-30

**Authors:** Amanda S. Mota, Anderson B. de Lima, Thayana Lucy F. Albuquerque, Tiago S. Silveira, José Luiz M. do Nascimento, Joyce Kelly R. da Silva, Alcy F. Ribeiro, José Guilherme S. Maia, Gilmara N. T. Bastos

**Affiliations:** 1 Laboratório de Neuroinflamação, Universidade Federal do Pará (UFPA), 66075-900 Belém, PA, Brazil; E-Mails: amanda.sodre.mota@gmail.com (A.S.M.); andersonbentes@uol.com.br (A.B.L.); thayanalucy@gmail.com (T.L.F.A.); 2 Programa de Pós-Graduação em Biotecnologia, Universidade Federal do Pará (UFPA), 66075-900 Belém, PA, Brazil; E-Mail: silveira.t@gmail.com; 3 Laboratório de Neuroquímica Molecular e Celular, Universidade Federal do Pará (UFPA), 66075-900 Belém, PA, Brazil; E-Mail: jlmn@ufpa.br; 4 Instituto de Ciências Exatas e Naturais, Universidade Federal do Sul e Sudeste do Pará (UNIFESSPA), 68507-590 Marabá, PA, Brazil; E-Mail: favacho@ufpa.br; 5 Programa de Pós-Graduação em Química, Universidade Federal do Pará (UFPA), 66075-900 Belém, PA, Brazil; 6 Programa de Pós-Graduação em Recursos Naturais, Universidade Federal do Oeste do Pará (UFOPA), 68035-110 Santarém, PA, Brazil

**Keywords:** *Plukenetia polyadenia*, Euphorbiaceae, fatty oil, linoleic and α-linolenic acids, antinociception activity, toxicity

## Abstract

Seed oil (Pp-oil) of *Plukenetia polyadenia* is used by native people of the Brazilian Amazon against arthritis and rheumatism, spreading it on the arms and legs to reduce the pain and inflammation. Pp-oil was obtained by pressing dried seeds at room temperature to give a 47.0% yield of oil. It was then subjected to fatty acid composition analysis. The principal fatty acids were linoleic acid (46.5%), α-linolenic acid (34.4%) and oleic acid (13.9%). Then, it was evaluated for its antinociceptive activity in mice, using the acetic acid-induced abdominal writhing, hot plate and formalin test models. Additionally, its toxicity was determined. The Pp-oil proved to have no toxicological effects, showing dose-dependent antinociceptive effect under chemical stimulation. At oral doses of 25–100 mg/kg, Pp-oil significantly reduced the abdominal writhes in the writhing test. A higher oral dose of 200 mg/kg did not induce alterations in the latency time of the hot plate test when compared to the control, suggesting an analgesic activity of peripheral origin. At oral doses of 50 and 100 mg/kg, the Pp-oil significantly reduced the second phase of the algic stimulus in the formalin test. In addition, the antinociception of Pp-oil was reversed by naloxone in the evaluation of its mechanism of action. Therefore, the Pp-oil proved to be safe at very high doses and to show significant analgesic properties. The role of Pp-oil is still being investigated with respect the mechanism of action, but the results suggest that opiod receptors could be involved in the antinociception action observed for the oil of *P. polyadenia.*

## 1. Introduction

*Plukenetia *L. is a neotropical genus of nineteen species belonging to the Euphorbiaceae. *Plukenetia polyadenia *Müll. Arg. [syn. *Elaeophora polyadenia *(Müll. Arg.) Ducke, *Elaeophora abutaefolia *Ducke and *Plukenetia abutaefolia *(Ducke) Pax & K. Hoffm.] [1] is a liana that climbs the canopy of tall trees, known as “compadre-de-azeite”. It grows in the wet lowland forests and is widespread in the the Amazon basins of the Guyanas, Venezuela, Ecuador, Peru, Bolivia and Brazil [2].

Some of the most common manifestations of diseases that affect millions of people worldwide are pain and inflammation. The dependence of the Amazon rural population with respect to medicinal plants is cultural, and the traditional medicine practitioners have used plants for their health care, particularly in the riparian communities. In Brazil, along the Amazon River and its tributaries, the seed oil of *P. polyadenia *(Pp-oil) is used by native people against arthritis and rheumatism. It is spread on the arms and legs to reduce the pain and inflammation. A single study of the seeds of *P. polyadenia * was previously reported, with the determination of its fatty acids composition and a preliminary toxicological evaluation [3]. 

Fatty acids are the primary precursors of important lipid mediators during the inflammatory process, such as arachidonic acid, prostaglandins, thromboxanes, and leukotrienes. Long chain n-3polyunsaturated fatty acids have been investigated for use in the treatment of inflammatory diseases such as rheumatoid arthritis, psoriasis, and ulcerative colitis, because of the presumed anti-inflammatory effects of these fatty acids [4,5]. The anti-inflammatory effects of long chain n-3 polyunsaturated fatty acids have been formerly attributed to changes in the production of prostaglandins and leukotrienes, although other studies have emphasized the reduced production of cytokines, as a possible mechanism [6].

The aim of this study was to analyze the fatty acids composition of Pp-oil and evaluate their antinociceptive activity and toxicity, based on its traditional use by the Amazon native population to reduce pain and inflammation in arthritis and rheumatism.

## 2. Results and Discussion

### 2.1. Oil-Composition

The percentage of the oil of *P. polyadenia* obtained by pressing the dried seeds at room temperature was 36.2%. This rate increases to 47.0%, considering the moisture content retained in the seeds, which was 30%. The extraction with Soxhlet showed a value of 56.2%, when used the *n*-hexane as solvent. Only the pressed dried seed oil was utilized in the experiments. The methylated fatty acids of Pp-oil were identified by GC/FID and GC-MS and are listed in Table 1. In addition, the Pp-oil was analyzed by ^1^H-NMR, and the assignments of their proton signals match well with the signals of the same methylated fatty acids already described in the literature [7,8].

**Table 1:** Methylated fatty acids of the oil of *Plukenetia polyadenia*.

Constituents	RI_Calc_	RI_Lit_	Oil %	Identification
Palmitic acid, methyl ester	1925	1921	2.9	GC, MS
Linoleic acid, methyl ester	2097	2092	46.5	GC, MS
α-Linolenic acid, methyl ester	2104	2098	34.4	GC, MS
Oleic acid, methyl ester	2112	2107	13.9	GC, MS
Stearic acid, methyl ester	2129	2124	1.5	GC, MS
Arachidonic acid, methyl ester	2279	2274	0.8	GC, MS
Total			100.0	GC, MS

RI_Calc_ = Retention index on DB-5 capillary column using *n*-alkanes (C8-C30) as standards; RI_Lit_ = Retention; index on similar capillary column described in the literature [9,10]; GC = Gas chromatography analysis: co-elution with methylated fatty acid standards; MS = Mass spectra.

### 2.2. Acute Toxicity (LD_50_)

Pp-oil did not demonstrate any behavior changes or mortality in mice at doses of 2000 mg/kg and 5000 mg/kg, during the three days of the experiment.

### 2.3. Clinical Chemistry and Histophatology

**Figure 1:**
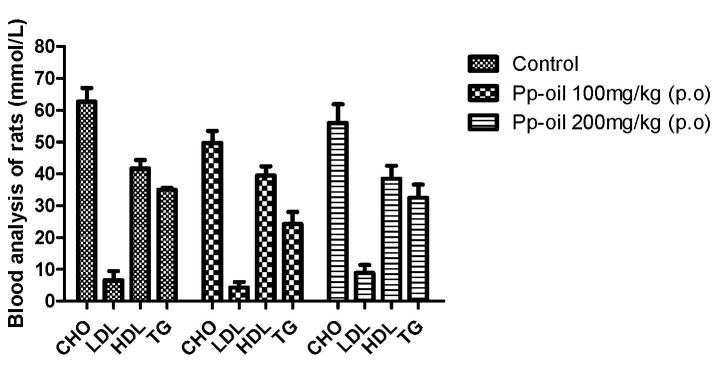
Clinical chemistry results for oral doses of 100 mg/kg and 200 mg/kg compared with control. Values are mean ± S.E.M (s.e.m.) and abbreviations: CHO, total cholesterol; LDL, low-density lipoprotein; HDL, high-density lipoprotein; TG, triglycerides.

**Figure 2:**
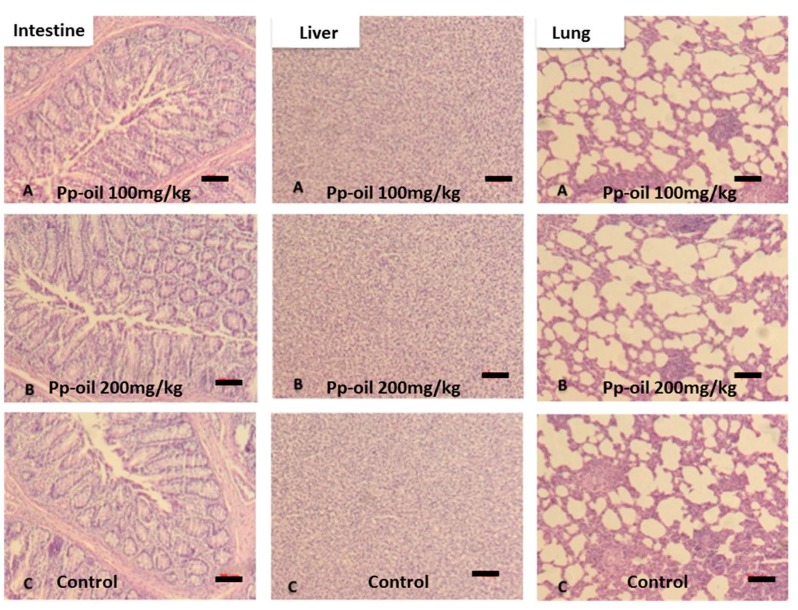
Histopathology of intestine, liver and lung: (**A**) 100 mg/kg; (**B**) 200 mg/kg and (**C**) control. Dark bar: 20 μm.

**Figure 3:**
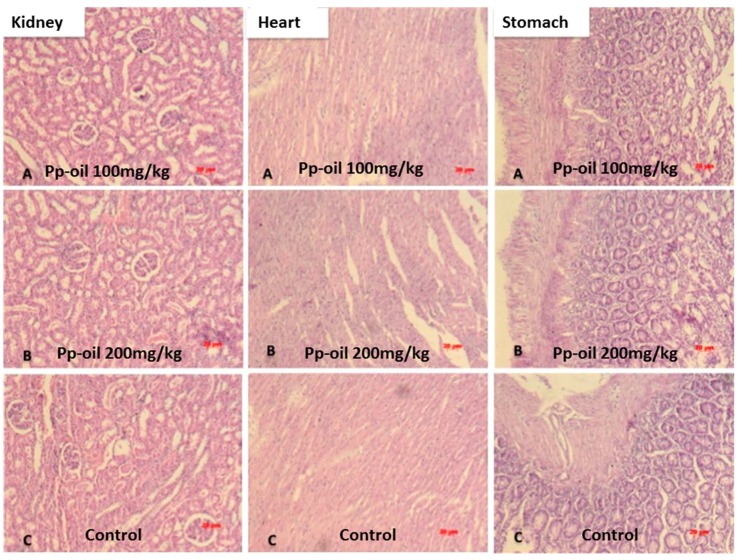
Histopathology of kidney, heart and stomach: (**A**) 100 mg/kg; (**B**) 200 mg/kg and (**C**) control. Dark bar: 20 μm.

The results of total cholesterol (CHO), low-density lipoprotein (LDL), high-density lipoprotein (HDL) and triglycerides (TG) of rats subjected to the experiment, using Pp-oil at doses of 100 mg/kg/day and 200 mg/kg/day, are shown in Figures 1. Values are shown as mean ± S.E.M (s.e.m.) and did not indicate chemical alterations in mice, when compared to the control. The histopathological examination can be seen in Figures 2 and Figures 3A–C, and also did not present any alteration in the morphology of the organs, at the used doses, when compared to the control.

### 2.4. Acetic Acid-Induced Writhing

**Figure 4:**
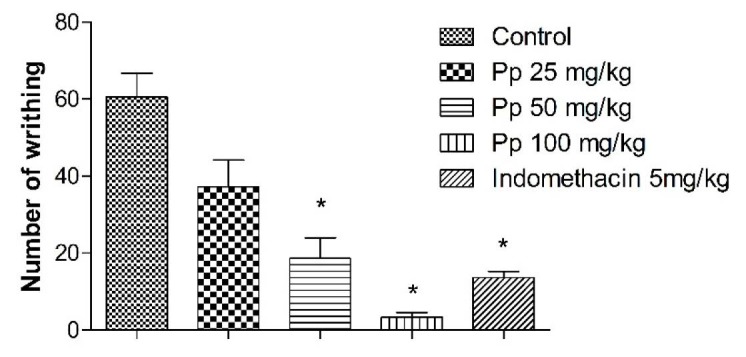
Effect of oral doses of Pp-oil on the nociception induced by intraperitoneal injection of acetic acid. Mean ± s.e.m., * Significantly different from the control (*p* < 0.05, F = 22.59, ANOVA, Student-Newman-Keuls *t*-test) at given a time.

Oral administration of the Pp-oil (25, 50 and 100 mg/kg) significantly decreased the number of writhes in mice induced by acetic acid, in comparison to the animals that received only the vehicle (Figure 4). These effects were dose-dependent.

### 2.5. Hot Plate Test

**Figure 5:**
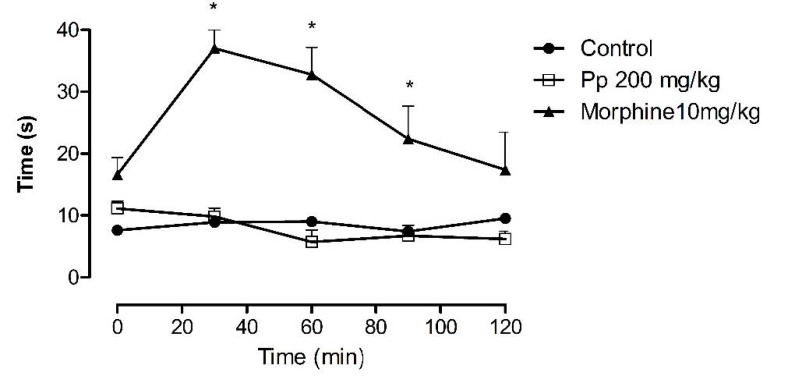
Hot plate test. Time-course of the effects of Pp-oil on thermal nociception. Abscissa time (min) after Pp-oil (oral), morphine (s.c.). Ordinate latency time (s) for the response to thermal stimulation (55 ± 0.5 °C, Mean ± s.e.m., *n* = 5) for each Pp-oil dose. * significantly different from the control (*p* < 0.05, F = 15.96; ANOVA, Dunnett’s *t*-test) at given a time.

Administration of Pp-oil dosed at 200 mg/kg did not induce alterations in the latency time in mice, when compared to the control (Figure 5).

### 2.6. Formalin Test

**Figure 6:**
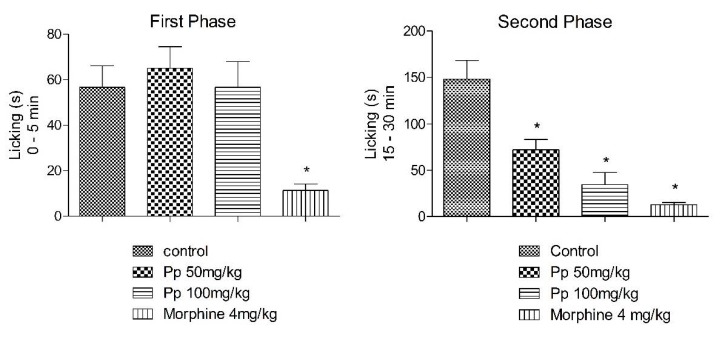
Formalin test: Phase 1 and Phase 2. Each group represents mean ± s.e.m. of 5 animals. * *p* < 0.05 when compared to the control value, F = 29.04 (ANOVA, Student-Newman Kuels’ test).

The Pp-oil (50 and 100 mg/kg) injected in mice 60 min before formalin showed a significant antinociception effect, reducing the liking time in the second phase (inflammatory) in a dose-dependent manner (Figure 6). 

### 2.7. Mechanism of Action

**Figure 7:**
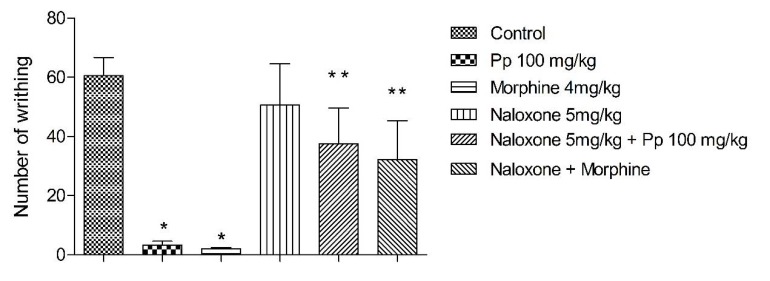
Mechanism of action. Each group represents mean ± s.e.m. of 5 animals. * *p* < 0.05 when compared to control value; ** *p* < 0.05 when compared to agonist plus antagonists * vs.* agonist alone, F = 4.855.

The pre-treatment of animals with naloxone showed a significant effect on the antinociception, during the writhing test. Naloxone reversed the effect caused by morphine and Pp-oil (Figure 7).

### 2.8. Discussion

The percentages of linoleic acid (LA, 18:2 n-6, omega-6, 46.5%) and α-linolenic acid (α-LNA, 18:3 n-3, omega-3, 34.4%) in Pp-oil were significant, in comparison to other fatty oils produced in the Brazilian Amazon region. Oleic acid (OA, 18:1 n-9, omega 9, 13.9%) was also present in the oil but with a lower content. Some oils from the Euphorbiaceae, such as *Ricinus communis* L. and *Jatropha curcas* L., contains triglycerides composed of fatty acids considered laxative and toxic [11,12]. It is not the case of Pp-oil, which is edible and composed only of fatty acids commonly found in vegetable oils used for cooking. Therefore, it was presumed that the administration of large quantities of PP-oil in mice would not elicit any toxic effects or death. This was confirmed by LD_50_, values.

The possibility of health benefits associated with the association of an omega-3 diet (polyunsaturated fatty acids, ω-3 PUFAs) have been described for several chronic conditions, including cardiovascular, neurodegenerative and neoplastic diseases [13]. Ample evidence has emerged over the last few years to show the critical role played by inflammation in the pathogenesis of these illnesses. Previously, they were not related to inflammation. Recently has been hypothesized that the effects of ω-3 PUFAs may be linked to their direct anti-inflammatory activity, as well its metabolites, eicosapentaenoic acid (EPA, 20:5) and docosahexaenoic acid (DHA, 22:6) [13]. n-6 PUFAs belong to another important family of PUFAs, also used in dietary and mainly concentrated in vegetable oils. Among the components of these two classes of PUFAs (n-3 and n-6), α-linolenic acid (α-LNA, 18:3 n-3) and linoleic acid (LA, 18:2 n-6) are considered essential for the human diet. The mammalian cells do not possess the desaturases able to place the double bonds specifically in n-3 and n-6 positions on the fatty acid carbon chain, differently from vegetable cells, along the synthetic pathway. Various synthesis steps produce n-6 PUFA arachidonic acid (AA, 20:4 n-6). It is highly represented in the membranes and is a precursor of the eicosanoids, the bioactive compounds that mediate the inflammatory process, as prostaglandins, thromboxanes, and leukotrienes.

The acute toxicity study was considerate as the initial phase of further sub-chronic studies. Doses up to 5000 mg/kg did not exhibit any mortality or any signs of toxicity after oral administration. This treatment may thus be considered as no-observed-adverse-effect level (NOAEL) [14]. The oral LD_50_ value in mice, when higher than 5000 mg/kg is described as class five (nontoxic substances) of the globally harmonized classified system for chemical substances and mixtures [15].

Pp-oil produced antinociception effects in the studied nociception models. The acetic acid-induced writhing reaction in mice has been primarily used as a screening tool for the assessment of analgesic or anti-inflammatory properties of new agents and is described as a typical model for visceral inflammatory pain [16]. The local irritation provoked by a test agent in the intraperitoneal cavity triggers a variety of mediators, such as bradykinin, substance P and the prostaglandins, especially, prostacyclin (PGI2), as well as some cytokines such as Interleukin-1 beta (IL-1β), tumor necrosis factor alpha (TNF-α), and Interleukin 8 (IL-8) [17,18,19]. These mediators can activate chemo-sensitive nociceptors that contribute to the onset of inflammatory pain. Pp-oil was able to reduce the writhing at doses of 25 and 50 mg/kg, suggesting that its antinociception effect could be related to inhibition of the release of mediators in response to acetic acid. The fatty oil could be inhibiting the production of a range of inflammatory proteins, including cyclooxygenase-2 (COX-2), inducible nitric oxide (NO) synthase, TNF-α, IL-1, IL-6, IL-8, and IL-12 in cultured endothelial cells, monocytes, macrophages, and dendritic cells. These inhibitory effects of long-chain n-3polyunsaturated fatty acids (PUFAs) seem to involve the decrease of IkB phosphorylation and reduce the activation of NFkB. These effects are associated with a reduction in the activation of the signaling proteins such as mitogen-activated protein kinases [20,21].

The hot plate test is a particular central antinociception test in which opioid agents exert their analgesic effects via supraspinal and spinal receptors. It is useful for the evaluation of centrally acting analgesics that are known to elevate the pain threshold of mice towards heat [22,23]. The Pp-oil did not alter the increase in latency for jumping or licking. These results are suggestive of an antinociception action of Pp-oil via a peripheral mechanism rather than a central acting mechanism.

The formalin test is believed to resemble clinical pain more closely in comparison with other tests that employ mechanical or thermal stimuli [16,24]. This test is a model of nociceptive response in two distinct phases, involving different mechanisms. The first phase (neurogenic pain) results from the direct chemical stimulation of myelinated and unmyelinated nociceptive afferent fibers, mainly C fibers, which can be suppressed by opioid analgesic drugs like morphine [25,26]. In the second phase, inflammatory mediators in the peripheral tissues, such as prostaglandins, serotonin, histamine and bradykinin, induce functional changes in the neurons, of the spinal dorsal horn that, in the long term, promote facilitation of synaptic transmission at the spinal level [27,28,29]. In this model, Pp-oil inhibited the licking response of mice in the second phase (Figure 4), suggesting this compound exerts its antinociception effects connected with peripheral mechanisms. The α-linolenic acid and the linoleic acid are known also as inhibitors of COX-1 and COX-2 [30,31], as well the morphine can promote a peripheral analgesia [32]. To this respect, naloxone, an opioid antagonist, was tested in the acetic acid writhing. It showed an influence on the antinociception action of Pp-oil (100 mg/kg, p.o.). This fact suggests the participation of the opioid system in the modulation of pain provoked by administration of Pp-oil.

The results of clinical chemistry and histopathology showed no significant difference between the two doses used and the control. Tissues were evaluated for the presence or absence of polymorphonuclear and mononuclear leukocyte; edema; apoptotic bodies and necrotic damage (see Figures 2,Figure 3).

## 3. Experimental Section

### 3.1. Drugs and Chemicals

The following drugs and chemicals were used: morphine (Laboratório Cristália, Itapira, SP, Brazil), indomethacin, sodium methoxide, boron trifluoride, *n*-alkanes (Sigma, St. Louis, MO, USA), naloxone (Laboratório Cristália), acetic acid, formalin, hexane, diethyl ether, methanol (Vetec, Duque de Caxias, RJ, Brazil).

### 3.2. Animals

Swiss male mice (20–25 g) were obtained from the Instituto Evandro Chagas (Belém, Pará, Brazil). They were randomly assigned to groups of ten animals and maintained in plastic boxes, with food and water *ad libitum*, under light and dark cycle (12 h, each). The room temperature was maintained at 22 ± 1 °C. The animals were acclimatized to the laboratory for at least 1 h before the experiments that were carried out between 8 h and 13 h in order to avoid circadian influence. All experiments reported in this study were conducted in accordance with current guidelines for the care of laboratory animals and ethical guidelines for investigation of experimental pain in conscious animals (CEPAE-UFPA 124-13). All efforts were made to minimize the number of animals used and their suffering.

### 3.3. Plant Material

The fruits of *P. polyadenia* were collected in a lowland area near of the Guamá River, and the municipality of Santa Isabel do Pará, Pará, Brazil, during the rainy season, April 2011. The plant was identified and deposited (MG 170411) in the Herbarium of Emílio Goeldi Museum, Belém, Pará, Brazil. The four seeds of the fruit were separated, dried at room temperature, then grinded and submitted to the oil extraction using an Expeller type press and a Soxhlet extractor. The moisture content of the seeds was calculated by drying in an electric oven until constant weight. The oil extracted from the pressed dried seeds was coded as Pp-oil and utilized in the experiments.

### 3.4. Fatty Acids Esterification

The Pp-oil (100 mg) was placed in a test tube (20 cm) with stopper. A methanol solution (3 mL) of sodium methoxide (3%) was added. The mixture was heated in a water bath at boiling temperature for 3 min. After, a methanol solution (3 mL) of boron trifluoride (10%) was added. The mixture was again heated in a water bath for other 3 min. The mixture was extracted with hexane (1 mL) and diethyl ether (2 mL) in a volumetric flask (50 mL) containing distilled water. The organic layer was separated to a vial (5 mL), and the solvent evaporated. The Pp-oil esterification was done in duplicate [33,34].

### 3.5. Oil-Composition Analysis

The Pp-oil was analyzed in a THERMO DSQ II GC-MS instrument, under the following conditions: DB-5ms (30 m × 0.25 mm; 0.25 mm film thickness) fused-silica capillary column; programmed temperature: 60–240 °C (3 °C/min); injector temperature: 250 °C; carrier gas: helium, adjusted to a linear velocity of 32 cm/s (measured at 100 °C); injection type: splitless (2 mL of a 1:1000 hexane solution); split flow was adjusted to yield a 20:1 ratio; septum sweep was a constant 10 mL/min; EIMS: electron energy, 70 eV; temperature of ion source and connection parts: 200 °C. The quantitative data regarding the methylated fatty acids were obtained by peak area normalization using a FOCUS GC/FID operated under similar conditions of the GC-MS, except for the carrier gas, which was nitrogen. The retention index was calculated for all the constituents using a homologous series of *n*-alkanes (C_8_–C_30_, Sigma–Aldrich, St. Louis, MO, USA). Individual components were identified by comparison of both mass spectrum and GC retention data with authentic compounds which were previously analyzed and stored in a private library, as well as with the aid of commercial libraries containing retention indices and mass spectra of methylated fatty acids [9,10]. The standards of methylated fatty acids were purchased from Sigma-Aldrich Brazil.

### 3.6. NMR Analysis of Pp-Oil

The ^1^H-NMR spectrum was obtained in a Varian Mercury NMR instrument at 300 MHz, using CDCl_3_ as solvent. 

### 3.7. Acute Toxicity (LD_50_)

Sixty male mice were used, aged 47 ± 2 days, body mass index between 20 and 38 g. They were divided into five groups of twelve. Each group fasted for 12 h received oral doses of Pp-oil between 2000 to 5000 mg/kg according to the body weight of each animal. Initially, the animals were observed for four h and placed in cages with food and water *ad libitum* and, then, kept for an additional 48 h. A separate group of animals was used as a control group, with regular food and water. The experiment aimed to observe the number of deaths in each group relative to the total number of animals treated with the Pp-oil. The toxicological effect was based on mortality, which was expressed as the medium lethal dose (LD_50_) [35,36].

### 3.8. 30-Day Chronic Oral Toxicity (Experimental Design)

This study was based on the enhanced OECD test, guideline 407 [37]. Briefly, rats at seven weeks of age were weighed and randomly assigned to four groups, with ten males and ten females in each cluster. Pp-oil was administered to the rats once daily by oral gavages at doses of zero (control), 100 and 200 mg/kg/day for at least 28 days, at a dosing volume of 5 mL/kg body weight. All animals were killed by exsanguinations under pentobarbital anesthesia between study days 31 and 32, and were autopsied. Blood samples for clinical chemistry were obtained from the abdominal aorta prior to necropsy.

### 3.9. Histopathology

All animals were subject to necropsy. Kidney, heart, stomach, liver, lung and intestine were fixed by immersion in neutral buffered formalin (10%). The samples were embedded in paraffin wax. Histological sections were stained with hematoxylin and eosin. The tissues were examined using light microscopy.

### 3.10. Clinical Tests

Blood samples for the hematology and biochemistry evaluation were collected from the retro-orbital plexus under light anesthesia induced by CO_2_ inhalation. EDTA was used as an anticoagulant for the hematology samples in the routine blood test and lithium heparin for the blood biochemistry. Food was held for approximately 18 h before blood collection. The samples were collected early in the working day to reduce biological variation. The standard biochemistry tests were used to found out metabolism products. Serum biochemistry was performed using a spectrophotometer (Hitachi7180+ISE full automatic biochemical analyzer, Hitachi Ltd., Gaoke, Japan) for the following analyses: total cholesterol (CHO), low-density lipoprotein (LDL), high-density lipoprotein (HDL) and triglycerides (TG). 

### 3.11. Antinociceptive Activity

#### 3.11.1. Acetic Acid-Induced Writhing in Mice

To evaluate the possible peripheral effects of the Pp-oil as analgesic, the writhing test by acetic acid was performed in mice [38]. Groups of five mice were fasted overnight prior to starting the experiment while given free access to water. The Pp-oil (25, 50 and 100 mg/kg), indomethacin (5 mg/kg) or equivalent volumes of vehicle (0.9% saline plus 1% Tween 80) were orally administered in mice 60 min prior to the acetic acid injection (0.6%). Indomethacin is a well-known peripheral analgesic drug, and it was used as a positive control. Then, 10 min after the acetic acid injection, the mice were placed in an observation box, and the writhing number was counted for 30 min.

#### 3.11.2. Hot Plate Test

Mice were pre-selected on the hot plate at 55 ± 0.5 °C. Animals when showing a reaction time (latency for licking the hind feet or jumping) greater than 20 s were discarded. Then, the selected mice were treated with vehicle (saline), Pp-oil (50, 100 and 200 mg/kg, p.o. via) or morphine (10 mg/kg, s.c.). The reaction time for each mouse was determined on the hot plate, before and after drug administration, at intervals of 30 min. A total period of 45 s was followed while measuring reaction time [39].

#### 3.11.3. Formalin Test

The formalin test was carried out according Hunskaar and Hole (1987) [40]. Formalin (20 μL, 1%) was administered to mice via intraplantar route and immediately the licking time was registered for 5 min (first phase, neurogenic). Fifteen minutes after beginning the experiment (second phase, inflammatory), the licking time was recorded for another 15 min. The animals were pre-treated with morphine (4 mg/kg, s.c.) to assess the possible participation of the opioid system in the antinociception effect, 15 min before administration of the Pp-oil (50 and 100 mg/kg, p.o.) or vehicle (0.9% NaCl, 10 mL/kg, p.o.). The algic responses caused by the first and the second phase of the formalin test were recorded 60 min after drug and vehicle administration. The other mice group received morphine 30 min before the formalin injection.

#### 3.11.4. Evaluation of the Mechanism of Action

Mice were pre-treated with naloxone (5 mg/kg, i.p.) to assess the possible participation of the opioid system in the antinociception effect, 30 min before the Pp-oil administration (100 mg/kg, p.o.), and morphine (4 mg/kg, s.c.) 15 min before the Pp-oil administration. The nociception response was evaluated by the acetic acid-induced contortions, and the evaluation of the mechanism of action was determined by the reversion of the antinociception effect in the Pp-oil.

### 3.12. Statistical Analyses

Results are expressed as mean ± S.E.M. Statistical evaluation were made using ANOVA followed by Student-Newman-Keuls or Dunn’s test, and the values were considered significantly different when *p *< 0.05.

## 4. Conclusions

In summary, at the oral doses tested, Pp-oil can be considered safe as it did not exhibit any lethality or adverse changes in the general behavior in the acute and sub-chronic toxicity studies in mice. It was demonstrated that Pp-oil exhibited dose-related antinociception when assessed in a chemical model, but not in thermal models of nociception in mice. Pp-oil has analgesic activity, which is probably of peripheral origin, according to the tests employed. The mechanisms involved were not yet completely investigated, although it seems that the opioid receptors could be involved in the antinociception action of the Pp-oil.

*Sample Availability*: Samples of fatty oil and pure compounds are available with the authors.
